# Novel advanced imaging techniques for cerebral oedema

**DOI:** 10.3389/fneur.2024.1321424

**Published:** 2024-01-31

**Authors:** Jenny Pham, Felix C. Ng

**Affiliations:** ^1^Department of Radiology, Royal Melbourne Hospital, Parkville, VIC, Australia; ^2^Department of Neurology, Royal Melbourne Hospital, Parkville, VIC, Australia; ^3^Department of Neurology, Austin Health, Heidelberg, VIC, Australia; ^4^Department of Medicine at Royal Melbourne Hospital, Melbourne Brain Centre, University of Melbourne, Parkville, VIC, Australia

**Keywords:** infarction, edema, imaging, net water uptake, stroke, malignant infarct

## Abstract

Cerebral oedema following acute ischemic infarction has been correlated with poor functional outcomes and is the driving mechanism of malignant infarction. Measurements of midline shift and qualitative assessment for herniation are currently the main CT indicators for cerebral oedema but have limited sensitivity for small cortical infarcts and are typically a delayed sign. In contrast, diffusion-weighted (DWI) or T2-weighted magnetic resonance imaging (MRI) are highly sensitive but are significantly less accessible. Due to the need for early quantification of cerebral oedema, several novel imaging biomarkers have been proposed. Based on neuroanatomical shift secondary to space-occupying oedema, measures such as relative hemispheric volume and cerebrospinal fluid displacement are correlated with poor outcomes. In contrast, other imaging biometrics, such as net water uptake, T2 relaxometry and blood brain barrier permeability, reflect intrinsic tissue changes from the influx of fluid into the ischemic region. This review aims to discuss quantification of cerebral oedema using current and developing advanced imaging techniques, and their role in predicting clinical outcomes.

## Introduction

Malignant infarction is a significant driver of mortality from ischemic stroke. Supported by the Stroke Treatment Academic Industry Roundtable (STAIR) X recommendations, there has been a growing interest in biomarkers to measure cerebral oedema to predict clinical outcome and assess cytoprotective treatment effect (1).

**Figure 1:**
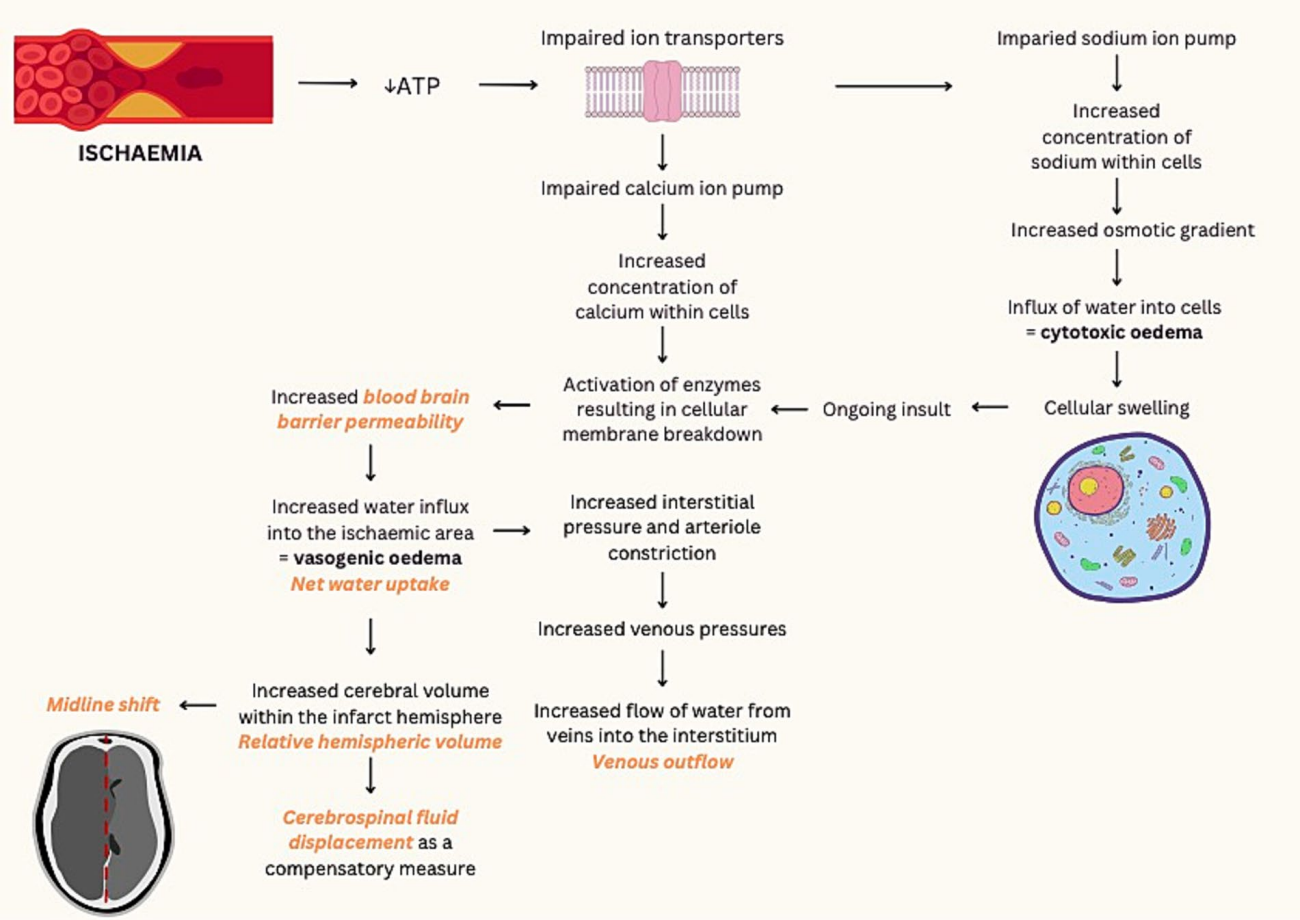
Pathological processes of ischemic infarction and associated imaging techniques. ATP, adenosine triphosphate. Imaging techniques demonstrated in orange text (2–6).

Within minutes of arterial occlusion, ion transporter function is impaired and osmotically active molecules move from the interstitium to the intracellular compartment. Consequently, cytotoxic oedema results in cellular swelling (Figure 1). With new gradients of sodium ions across the blood brain barrier, ionic oedema progresses and further promotes the influx of water into the ischemic area. Maintained hypoxia results in irreversible cellular damage secondary to the breakdown of cellular membranes, leading to the impairment of endothelial junctions and vasogenic oedema (2).

Radiological appearance of cerebral oedema in ischemic stroke may be assessed via two main methods: macroscopic neuroanatomical distortion and intrinsic cerebral tissue changes. In current clinical practices, anatomical shift or measuring the observable mass effect is the conventional approach to assess cerebral oedema. Quantitative measurement of midline shift is typically used as a reference standard, while qualitative measures such as the presence of ventricular distortion and herniation are important secondary signs of cerebral oedema that can be readily evaluated by clinicians for clinical decision making. In contrast, approaches to assess intrinsic tissue changes seek to assess the primary pathological processes at a tissue level. These are predominantly measures applied in stroke research and includes measuring hypoattenuation on CT imaging and T2 changes on magnetic resonance imaging (Table 1).

**Table 1:** Imaging metrics for cerebral oedema.

	Measurement	Pathology	Imaging analysis	Clinical applicability	Key studies
Neuroanatomical shift	Midline shift	Space-occupying cerebral oedema within a fixed volume results in displacement of structures	Deviation of the septum pellucidum between a line drawn between the anterior and posterior attachment of falx cerebri	Reference standard for cerebral oedema	Ropper (7) Hofmeijer et al. (8)
	Relative hemispheric volume	Increased tissue volume due to water influx	Ratio of slice-by-slice segmentation of the ischemic hemisphere to contralateral side, excluding major sulci, cisterns and ventricles	Strong correlation with midline shift. In animal models, it has been correlated with absolute water volume	Gerriets et al. (9) Ng et al. (10)
	Cerebrospinal fluid displacement	Displacement of CSF as a compensatory mechanism to counteract oedema-related brain expansion	Slice-by-slice CT segmentation of CSF spaces. Used as a ratio with contralateral hemisphere or intracranial size	May allow for early identification of cerebral oedema without need for identification of infarct or hypodensity on baseline imaging	Minnerup et al. (11) Kauw et al. (12) Dhar et al. (13)
Tissue-based properties	Net water uptake	Cytotoxic and vasogenic oedema result in water uptake within the ischemic lesion, reflected by hypoattenuation of the infarcted area	Infarct lesion is outlined using CT perfusion imaging or ASPECTS region HU of infarct area compared to contralateral parenchyma (1-ROI HU/contralateral ROI HU) × 100	Identify patients presenting within 4.5 h Used to predict poor outcomes Investigated as a tool to assess post-treatment follow up imaging, however this is not yet validated	Minnerup et al. (4) Broocks et al. (14) Nawabi et al. (15) Broocks et al. (16) Ng et al. (10)
	T2 relaxometry	Water influx results in increase of T2-weighted hyperintensity	Quantification of lesion voxel change with T2-weighted imaging	May aid to predict stroke onset in patients with poor cerebral blood flow Lack of data of serial assessment and correlation to clinical outcomes	Wouters et al. (17) Wang et al. (18)
	Blood brain barrier permeability	Cellular damage due ischemia results in increased permeability	Analysis of contrast movement from intravascular to extravascular compartment on CT perfusion via microvascular permeability metrics or MRI using Gd-DTPA	Correlation with hemorrhagic transformation and poor outcomes Lack of agreement of approach for measurement and assumption of unidirectional flow	Horsch et al. (19) Dankbaar et al. (20) Bektas et al. (21) Ng et al. (22)
Other surrogate markers	Venous outflow profile	Increased interstitial pressure and resistance of downstream arterioles, resulting in increased venous pressure	Assessment of the patency of cortical veins on CT imaging. Use of scoring systems such as COVES and PRECISE	Favorable profiles associated with reduced oedema progression rate and functional outcome Limited by anatomical variability and non-standardized imaging protocols	Van Horn et al. (23) Parthasarathy et al. (24)

Early identification and quantification of cerebral oedema is critical in assessing candidates for decompressive surgery due to risk of malignant infarction (25, 26). In this review, we aim to discuss quantification of cerebral oedema using current and developing advanced imaging techniques, and their role in predicting clinical outcome.

## Neuroanatomical shift

### Midline shift

Midline shift is typically defined as the deviation of the septum pellucidum from a line drawn between the anterior and posterior attachments of the falx cerebri. Reflective of space-occupying cerebral oedema in anterior circulation hemispheric stroke, rapid increase of midline shift is suggestive of malignant infarction and is correlated with mortality (7, 8, 27). Midline shift is the established reference standard for cerebral oedema both in research and clinical practice and in stroke, and is widely applicable to all other cerebral pathology in the cerebral hemisphere (28, 29). This has been further supported by a recent prospective multicenter study by Wu et al. (26) that demonstrated the association of midline shift with malignant infarction in a large cohort of 2,123 patients. The main advantage of midline shift is that it can be easily and rapidly applied without the need for dedicated analysis software or prior training. However, it is typically a delayed sign with limited sensitivity for small cortical infarcts that is anatomical remote for midline structures.

### Relative hemispheric volume

Relative hemispheric volume (rHV) quantifies the three-dimensional volumetric expansion of the ischemic hemisphere compared to the contralateral hemisphere. It involves segmentation of the ipsilateral and contralateral cerebral hemispheres, with exclusion of the major sulci, cisterns and ventricles. In animal models, relative hemispheric volume has been correlated with absolute water volume (9, 30). However this has not been validated in humans. A recent analysis of RHAPSODY patients (*n* = 65) by Schleicher et al. (31), demonstrated a strong correlation between measurements of rHV, midline shift and change in cerebrospinal fluid volume on CT imaging at day 2. In contrast, there is a lack of correlation between these anatomical markers of cerebral oedema with NWU (10, 31). Relative hemispheric volume is an effective measure of tissue volume and is shown to be superior to midline shift in predicting clinical outcome (10, 32–34). Currently, rHV has limited clinical application due to the time consuming nature of its measurement. With the development of automated algorithms, analogous to that in cerebrospinal fluid (CSF) displacement measurements, efficiency of rHV may improve (35).

### Cerebrospinal fluid displacement

Displacement of CSF is one of the earliest compensatory mechanisms to counteract oedema-related brain expansion (3). CSF displacement as a quantitative metric has been shown to correlate with other measures of cerebral oedema such as midline shift and has been demonstrated to be an independent predictor malignant middle cerebral artery (MCA) infarction (36, 37).

In an early cohort study, Minnerup et al. (11) assessed the ratio of infarct volume to CSF volume on admission multimodal CT imaging for 52 patients, using semiautomated slice-by-slice segmentation, and divided this by intracranial volume to account for cranial size variability. Larger infarct volume to CSF ratio was found to be associated with malignant MCA infarction. These findings were supported by multiple subsequent studies, which demonstrated that CSF volume is an independent predictor of malignant infarction (12, 36, 37).

In 2018, Dhar et al. (35) developed an automated algorithm using machine learning to segment CSF spaces on non-contrast CT brain studies. They subsequently analyzed the change in CSF volume at baseline and within 12 h of presentation in 474 patients, of which only 74 patients demonstrated midline shift. They found that odds of midline shift development doubled for every 10% reduction in CSF in the first 24 h. Furthermore, this change in CSF volume was associated with functional outcomes (mRS) independent of stroke severity.

Similar to previous studies that used CSF volume ratios to mitigate anatomical variability (11, 37), Dhar et al. (13) turned to analysis of hemispheric CSF ratio, that is the CSF volume of the affected hemisphere compared to the non-affected hemisphere. In this study, they analyzed 924 CT studies performed within 96 h of stroke onset and used follow-up imaging (*n* = 737) to assess cerebral oedema development. In multivariable analysis, they found that lower CSF ratio at 24 h was associated with higher NIHSS, lower ASPECTS and lower baseline CSF volume. Moreover, they noted that a hemispheric CSF ratio below 0.50 had a 90% sensitivity and 82% specificity to identify patients who developed malignant infarction.

There are several strengths of using CSF volumes as a metric of cerebral oedema. Via semiautomated processes or machine learning algorithms, quantitative analysis of CSF volumes or ratios may allow for early identification of cerebral oedema without need for identification of infarct or hypodensity on baseline imaging, unlike midline shift, ASPECT scores and NWU. In particular, use of CSF ratios on admission imaging reduces confounding anatomical variability and need for serial imaging (13).

While there are clear strengths in this method, including its accessibility, correlation with current measures of oedema and prediction of clinical outcome, there are cohorts for which CSF volumetric imaging has limited value. These include patients with old infarcts and encephalomalacia resulting in baseline asymmetrical CSF volume; images with significant artifact; or in patients with baseline midline shift, for which shifted ventricles may be misregistered (13).

## Tissue-based properties

### Net water uptake

Pathological cellular uptake of extracellular fluid into the ischemic tissue results in lesion hypodensity on CT imaging (14). Net water uptake is a Hounsfield-unit (HU) based metric, which leverages the lower attenuation coefficient of the oedematous infarct tissue and is calculated by comparing HU of the infarct area to that of the contralateral parenchyma [(1-ROI HU/contralateral ROI HU) × 100] (4, 38). Infarct area is typically outlined using CT perfusion imaging, although an alternative using affected Alberta Stroke Program Early CT Score (ASPECTS) region has been proposed by Minnerup et al. (4), Cheng et al. (39), and Broocks et al. (40).

#### Early oedema on baseline imaging

Quantification of cerebral oedema may assist in guiding acute reperfusion management of patients with unknown time of onset, who represent approximately 25% of all ischemic strokes (40). Due to the progressive nature of cerebral oedema following large vessel occlusion, multiple groups have used CT perfusion or ASPECTS-derived NWU calculation to generate cut off values that would enable identification of patients presenting within 4.5 h to guide active treatment (4, 39). This concept of using pre-treatment hypodensity as a surrogate of tissue oedema was first proposed in a multicenter study of 147 patients by Minnerup et al. (4) identified a NWU cut off value of 11.5% with 100% specificity and 98.6% sensitivity. Broocks et al. (40) then compared this cut off value of 11.5% to diffusion weighted imaging/fluid attenuation inversion recovery (DWI/FLAIR) mismatch and showed that both methods are comparable. Although limited by sample size of 50 patients, they demonstrated that quantitative NWU may be a reliable indicator for lesion age in the first few hours of symptom onset. However, they note that beyond this time there is patient variability, supporting findings of Nawabi et al. (15) who proposed the idea of a “tissue clock.”

Nawabi et al. (15) proposed the idea that early elevated levels of NWU, that is cerebral oedema, is predictive of poor outcomes despite presenting within the traditionally accepted “time clock.” This discrepancy between “tissue clock” and “time clock” is supported by several studies who have used NWU as a quantitative tool to predict clinical outcome. Through analysis of admission imaging, several groups have demonstrated that NWU values greater than 10% or 12.7% is suggestive of poor functional outcomes following MCA infarcts (14, 15).

#### Post-stroke malignant oedema assessment and prediction

Unsurprisingly, NWU on admission CT has also been shown to be associated with the development of post-stroke malignant oedema. In their analysis, Broocks et al. (14) found that the probability of malignant infarct was significantly associated with early infarct volume and NWU, with 1% increase in NWU being associated with 1.27 increased risk of malignant infarction. In a separate study, this group also found that NWU also predicted malignant oedema in the posterior circulation (41). The strength of NWU as a biomarker of cerebral oedema on pretreatment imaging is likewise shown when using ASPECTS-derived NWU, with ASPECTS-NWU being an independent predictor of neurologic outcome at 90 days, even in subgroup analysis of patients with ASPECTS less than 5 (42–44). This presents the notion that patients with low ASPECTS, traditionally thought to have a poor outcome, may still benefit from endovascular thrombectomy if NWU remains relatively normal or is only mildly deranged (45, 46).

#### Post-treatment follow-up imaging

NWU has been increasingly applied to post-treatment follow-up imaging to study the treatment effect of endovascular thrombectomy on infarct evolution and cerebral oedema. Through analysis of early follow up non-contrast CT imaging post endovascular thrombectomy for anterior circulation infarcts, Broocks et al. (47, 48) found that NWU was double in persistent large vessel occlusion (16%) compared to patients with vessel recanalization (8%), and thus concluded that successful recanalization was associated with reduced progression of ischemic oedema. In another study, 2% of 184 patients demonstrated reversal of vasogenic oedema 24–48 h post-thrombectomy (16). Konduri et al. (49) extended analysis of oedema progression in ischemic lesions following intervention into the subacute time period. They demonstrated that cerebral oedema and infarct volume continued to increase, which was believed to be secondary to ischemic and reperfusion injury to the blood brain barrier. At 1 week, patients who had greater increase in NWU post-thrombectomy were associated with futile recanalization. Recently, in a multicenter observational study of patients from a prospective registry, Broocks et al. (50) proposed the use of NWU to stratify risk of futile recanalization for patients with low ASPECTS. These findings suggest that NWU may improve prognostication post-thrombectomy compared to using infarct volume or ASPECTS as the sole prognostic marker (51, 52).

However, in a pooled patient analysis (*n* = 144), Ng et al. (10), demonstrated that NWU on non-contrast CT post-thrombectomy poorly correlated to midline shift and relative hemispheric volume. These volumetric measures of cerebral oedema were demonstrated to be superior to NWU as prognostic markers. Importantly, they found that NWU did not correlate with functional outcome at 90 days in the presence of hemorrhagic transformation or thrombectomy. These findings may be partially due to angiographic iodine retention, which may confound NWU measures which is dependent on Hounsfield-unit of the infarct tissue (32). Iodine extravasation may account for 27–84 percent of hyperdense lesions on CT post-thrombectomy at 24-h (53, 54). To investigate the impact of iodine retention, Steffen et al. (55) conducted a pilot retrospective single center case series of 10 patients following MCA infarcts using CT and dual energy CT (DECT) imaging. While no statistically significant difference were demonstrated in this small and likely underpowered cohort, notably 2 of 10 patients demonstrating more than 20% differences in NWU values between conventional CT and DECT, implying that the effect of iodine may affect NWU measures in a substantial proportion of patients. A subsequent larger multicenter study (*n* = 125) aimed to provide a more definitive analysis of the confounding effects of post-thrombectomy iodine on follow-up CT demonstrated that contrast-adjusted NWU values were significantly higher than non-adjusted values, and correlated with measures of cerebral oedema, such as midline shift and rHV (32). They demonstrated that even without visible hyperdense lesions on follow up imaging at 24 h, angiographic iodine contrast is retained in brain parenchyma.

In addition to these limitations, NWU measurement is technically challenging and may be confounded by hemorrhagic transformation which is highly common on post-treatment CT, potentially limiting the utility of NWU as a useful metric in follow-up scans and as a surrogate radiological outcome. Identification of infarcted tissue is required for precise density measurement, which may be subtle in the very early stages of stroke changes on non-contrast CT imaging. A majority of earlier studies on NWU were dependent on CT perfusion to delineate the ischemic lesion at baseline, which negates the primary advantage of NWU to assess for tissue injury at baseline without the need for contrast-enhanced imaging. Further investigation into these factors and consideration of contrast retention when evaluating NWU on post-treatment imaging needs to occur prior to application in the clinical setting.

### T2 relaxometry

In addition to ischemic stroke, quantitative hyperintensity on T2-weighted imaging due to intrinsic properties of water has been used to assess other neurological diseases such as peritumoral oedema, epilepsy and multiple sclerosis (56, 57). T2-weighted or FLAIR imaging has been used to estimate lesion age, with DWI/FLAIR mismatch being suggestive of onset within 4.5 h (58). With synthetic MRI imaging, quantification of lesion voxel change is possible with the added benefit of reduced T1 effect (59). In animal models, T2 relaxation time has been shown to linearly increase in ischemic tissue in the acute setting (60). This has provided the basis of quantitative evaluation of T2-weighted imaging.

T2 relaxation time has been demonstrated to be affected by cerebral blood flow and collateral status (17, 18). Wang et al. (18) demonstrated that in patients with poor cerebral blood flow, T2 relaxometry correlated with stroke onset time, with no significant correlation in the group with good cerebral blood flow. However, as T2 relaxometry is highly specific to tissue composition, there are limitations to this technique due to variability with anatomical location (59). For this reason, alternative methods to measure T2-weighted signal have been sought to minimize confounding variables, such as the spherical reference method and the mirror method (60–62). Although it is known that DWI lesions may grow with time, few studies have assessed serial T2 relaxometry imaging and its relationship with outcome.

### Blood brain barrier permeability

In the setting of ischemic stroke, tissue blood brain barrier permeability is elevated in hypoperfused areas secondary to cellular damage, which consequently leads to inappropriate influx of blood and extracellular fluid into ischemic tissue and cerebral oedema (5). Although collateral arterial supply may reduce blood brain barrier permeability, loss of integrity has been demonstrated to occur in the first few hours of ischemia, as fast as 20 min in rodent animal models (19–21, 63–66).

On CT imaging, blood brain barrier permeability is measured using CT perfusion studies via analysis of movement of contrast from the intravascular to extravascular compartment within the hypoperfused area. It involves using the Patlek method to calculate microvascular permeability metrics, transendothelial transfer constant (kPS) or permeability surface area product (PS, ml/min/100 g), based on time-density curves for each pixel (19, 21). The use of relative permeability-surface area product (rPS = PS/CBF × 100) is of interest due to the correction for the potential influence of cerebral blood flow (19, 20, 67). In normal parenchyma, the permeability product is null for large molecules, such as iodine contrast.

Several studies have measured blood brain barrier permeability on MRI imaging using gadolinium-diethylene triamine pentaacetic acid (Gd-DTPA), which does not cross the blood brain barrier (66, 68). Extravasation of contrast into CSF reduces the T1 relaxation of CSF, resulting in hyperintensity of the CSF space on T1-or T2*-weighted imaging (66, 68). As such, T1-weighted dynamic contrast-enhanced, FLAIR and T2*-weighted dynamic susceptibility contrast sequences have been used for perfusion-weighted imaging (68–70). As in CT perfusion imaging, change of pixel intensity can be measured following contrast injection and used as a measurement of permeability (22, 59, 61, 66, 71).

Blood brain barrier permeability has been correlated with hemorrhagic transformation (66, 72–75). In small retrospective studies, Lin et al. (72) and Hom et al. (73) found that in patients with high PS within the infarct area, who then received tissue plasminogen activator had an increased risk of hemorrhagic transformation. In their receiver operating characteristic curve analysis of 32 patients, Hom et al. (73) demonstrated that blood brain barrier permeability measurements above the threshold of 7 mL/100 g/min predicted symptomatic hemorrhagic transformation and malignant oedema with 100% sensitivity and 79% specificity. They postulated that tissue plasminogen activator upregulates matrix metalloproteinases, which damages blood brain barrier integrity. Similarly, early blood brain barrier disruption measured with MRI imaging has been associated with hemorrhagic transformation and poor outcomes (66, 75). In a recent study analyzing MRI permeability and circulating inflammatory markers, Bani-Sadr et al. (70) found that in patients with onset of symptoms within 6 h of imaging (*n* = 72), blood brain barrier permeability was independently associated with increased matrix metalloproteinase-9 levels and larger ischemic core.

Interestingly, some studies suggest that blood brain barrier integrity may recover following reperfusion, thereby limiting cerebral oedema (68). Using dynamic susceptibility contrast MRI perfusion, reversible blood brain barrier permeability was found to correlate with increased reperfusion (22, 68). This supports the bi-phasic permeability hypothesis, in which there is an early reversible phase and a later irreversible phase (76).

There are several challenges in using blood brain barrier permeability as a measure of cerebral oedema. Foremost is the lack of agreement on an approach for measurement. There is debate as to whether first-pass contrast or delayed acquisition CT perfusion data is more accurate, with suggestion of first-pass data leading to overestimation of permeability (73, 77). On the other hand, delayed perfusion CT acquisitions involves greater radiation dose and risk of motion artifact. Moreover, critical assumption pertaining to this model is that of unidirectional flow across the blood brain barrier (19, 20). These limitations are echoed in MRI measurements with non-linear behavior of contrast and lack of fast imaging techniques.

## Other surrogate markers

### Venous outflow

With progressive cytotoxic oedema and water uptake in infarcted tissue, interstitial pressure and increased resistance of downstream or collateral arterioles may result. Resultant increase in venous pressure promotes fluid leakage into the infarcted tissue (6). Several studies have analyzed venous flow of deep and superficial veins to assess transition of blood through the ischemic territory. Unlike CT angiography of collateral arterial vessels, venous outflow imaging reflects both the microvascular inflow and outflow circuits (23, 78). Scoring systems have been developed to quantify venous drainage abnormality on CT angiography. The “Prognostic evaluation based on cortical vein score difference in stroke” (PRECISE) score compares the patency of both superficial and deep veins (the superficial cerebral vein, vein of Labbé, vein of Trolard, basal vein of Rosenthal, thalamostriate vein and internal cerebral veins) to the contralateral hemisphere (24, 79). In contrast, the cortical vein opacification score (COVES) quantifies opacification of the vein of Labbe, sphenoparietal sinus and superficial middle cerebral vein, with a score of zero being indicative of no filling (78).

Both scoring systems correlated with to NWU and may be used as a predictive factor for functional outcome (23, 24, 78–80). Patency of venous outflow vessels associated with the infarct region, deemed as favorable venous profiles, are associated with good function outcome, independent of other clinical factors including arterial collaterals, ASPECTS and NIHSS (23, 81, 82). In their multicenter retrospective study involving 728 patients, van Horn et al. (23) found that favorable venous profiles (COVES) on imaging prior to endovascular thrombectomy, NIHSS and time from onset to admission imaging were independently associated with reduced oedema progression rate. Conversely, unfavorable venous profiles have been linked with elevated NWU and may impact the risk benefit discussion of EVT, with studies suggesting that patients without venous opacification (COVES 0) did not benefit from treatment (78, 82).

However, there are limitations of using venous outflow profile scoring as an indirect measure of cerebral oedema. Predominantly, this lies in the anatomical variation of cortical venous structures between individuals. However, differences in imaging protocols also contribute to limited reliability of results, particularly in single phase CT angiography, whereby early timing of intravenous contrast bolus may lead to underestimation of venous opacification (24, 80). Several of the aforementioned studies excluded patients with inadequate timing, which may further contribute to bias of results (23, 78, 81, 82).

## Conclusion

Cerebral oedema following acute ischemic infarction is the driving mechanism of malignant infarction. Aiming to quantify cerebral oedema within the early stages of ischemic infarction, many novel imaging biomarkers are fundamentally based on pathophysiology of oedema at the cellular level. It is therefore unsurprising that correlations have been made between these imaging metrics to risk of malignant infarction and functional outcomes. However, there are many challenges that must be met prior to application to clinical practice, including the lack of consensus for measurement and anatomical variability. Despite this, these biomarkers have the potential to challenge current paradigms and ultimately evolve clinical management of ischemic stroke.

## Author contributions

JP: Writing – original draft, Writing – review & editing. FN: Conceptualization, Writing – review & editing, Supervision.
